# The Measurement of Ethnic Segregation in the Netherlands: Differences Between Administrative and Individualized Neighbourhoods

**DOI:** 10.1007/s10680-018-9479-z

**Published:** 2018-03-21

**Authors:** Bart Sleutjes, Helga A. G. de Valk, Jeroen Ooijevaar

**Affiliations:** 1 Netherlands Interdisciplinary Demographic Institute (NIDI)/KNAW/University of Groningen, The Hague, Netherlands; 2 Statistics Netherlands (CBS), The Hague, Netherlands

**Keywords:** Ethnic segregation, the Netherlands, Individualized neighbourhoods, Segregation measurement

## Abstract

The debate on residential segregation often focuses on the concentration of migrant groups in specific neighbourhoods and its presumed effects on, e.g. personal life chances and social inclusion. However, cross-regional and international comparisons of segregation are hampered by differences in the size and delineation of the spatial units that are used for its measurement: the Modifiable Areal Unit Problem. This paper therefore measures segregation for scalable ‘individualized neighbourhoods’, defined by a predefined number of closest neighbours instead of by administrative or statistical boundaries. This approach allows for measuring segregation levels and patterns across different spatial scales, ranging from the micro-scale (50 neighbours) to larger spatial areas (51,200 neighbours). Using population register data from the Netherlands, we study the segregation of four different migrant origin groups across individualized neighbourhoods at eleven spatial scales. Outcomes are compared to those found using administrative neighbourhoods. We are especially interested in how levels and patterns of segregation change with an increase in scale level. Our findings indicate that segregation levels and patterns are different across various spatial scales, and the most relevant spatial scale is also group-specific. Measuring segregation while using scalable individualized neighbourhoods seems an appropriate way to deal with both the multiscalar nature of segregation and the large within-district variety associated with it.

## Introduction

Ethnic segregation, the unequal distribution of migrant groups across space (Musterd 2005), is one of the most discussed issues with regard to migration settlement in both the USA and Europe. Settlement patterns of newly arriving immigrants as well as of those residing in the country already for longer periods are thought to influence social and economic integration, inter-ethnic contact and social cohesion in society (Pinkster 2008; Musterd 2011; Lichter et al. 2012). Although studies report ethnic segregation in many countries, there is still an ongoing debate on how it should be measured. Part of this discussion focuses on the most appropriate spatial units that should be used for its measurement. Traditionally, segregation is measured for ‘administrative neighbourhoods’ or units constructed for data collection (from here on: ‘statistical neighbourhoods’), with fixed borders that are generally drawn by authorities. International or regional comparisons of segregation patterns are affected by the different ways in which these units are defined, as well as by differences in their population size across and even within countries. This is referred to as the *Modifiable Areal Unit Problem* (MAUP) (Malmberg et al. 2011, 2014).

Another issue that is central in the recent debate on residential segregation is the role of spatial scale. A number of recent studies on residential segregation have argued that segregation is a multiscalar phenomenon. It is continuous across different spatial scales and should therefore ideally be measured at different spatial scales simultaneously, rather than in a static spatial setting (Fowler 2015; Clark et al. 2015; Jones et al. 2015). More recently it has been suggested to construct ‘individualized neighbourhoods’ to deal with MAUP and the issue of scale. Instead of fixed boundaries, these individualized neighbourhoods are based on an individual’s exact location and districts can be based on either a fixed distance radius (Reardon et al. 2008; Lee et al. 2008) or a predetermined number of nearest neighbours (Östh et al. 2014; Andersson and Malmberg 2015). The increased availability of grid data has stimulated this development which allows for comparisons across different spatial scales by varying the number of nearest neighbours or the distance radius from the same location. Previous studies in which individualized neighbourhoods were applied focused primarily on Sweden or Great Britain. In addition, with the exception of Van Ham et al. (2014) and Clark et al. (2015), the methodology has hardly been used to study ethnic segregation. But one could argue that also the concentration of migrant origin populations is continuous across various spatial scales, ranging from ethnic enclaves at the micro-level to concentrations encompassing entire metropolitan areas. Studies on ethnic segregation focusing on administrative or statistical districts already show large spatial variations. A multiscalar approach with scalable individualized neighbourhoods could help to unravel how these concentrations differ not only across, but also within and at the border of administrative or statistical districts.

This study explores to what extent using scalable individualized neighbourhoods provides a more nuanced picture of segregation compared to static administrative or statistical units in the Netherlands. This country is an excellent case to study these processes and the implications of different methods. The Dutch population is ethnically mixed, which allows disentangling specific patterns by migrant origin groups. Previous evidence showed that non-western migrants and their descendants in the Netherlands are not equally dispersed across the country and across the different administrative neighbourhoods of cities (Musterd and Van Kempen 2009; Hartog and Zorlu 2009; Das 2010). Besides aggregated data at the level of administrative neighbourhoods, boroughs and municipalities, the Netherlands also has rich population register data available, including geocoded address information that allows for constructing individualized measures.

The main aim of this study is to get better knowledge on the role of spatial scale for segregation measurement. In the first part of the analysis, segregation is defined according to the relative share of a certain migrant origin group within the total population of a specific spatial unit. Subsequently neighbourhood typologies are developed, based on the concentration of the four largest non-western migrant origin groups (Surinamese, Antilleans, Moroccans and Turkish) and the native population in the Netherlands. The dispersion of these neighbourhood types is illustrated by maps. In the second part of the analysis, we use another definition of segregation: the ‘isolation index’, which measures the degree to which individuals are potentially exposed to either members of their own group (isolation) or another group (interaction) (Nijkamp and Poot 2015). Here the focus is on the Amsterdam Metropolitan Area. In both parts, results are compared between administrative units at three static spatial scales on the one hand and eleven scalable individualized units on the other.

## Ethnic Segregation Patterns in the Netherlands

### Migration History

Although the Netherlands has been an immigration country ever since the seventeenth century, the current stock of migrants was formed mainly by migration flows since the Second World War. During the past decades, the independence of former colonies, guest worker recruitment and family reunification have resulted in an ethnically diverse society. On 1 January 2012, the Netherlands had a total migrant population of 3,427,000 persons. In this paper, we focus on four of the largest migrant origin groups: Moroccans (363,000 on January 1 2012), Turks (393,000), Surinamese (347,000) and Antilleans (144,000) (Statistics Netherlands 2012).

In the 1960s, the recruiting of ‘guest workers’ resulted in large flows of migrants: first from Spain and Italy, but especially from the rural parts of Turkey and Morocco. Although their residence was initially thought to be temporary, many stayed and were later followed by their families. However, the position on the labour market of the Turkish and Moroccan migrants, and the first generation of ‘labour migrants’ has been difficult. They were generally recruited into low-level jobs, have a limited level of education and were faced with language difficulties and a lack of integration programmes (Vermeulen and Penninx 2000; Van Mol and De Valk 2016).

In the 1970s, the independence of the former colony of Surinam resulted in a large flow of Surinamese migrants to the Netherlands (Vermeulen and Penninx 2000). This group was followed by a smaller flow of migrants from the islands of the Netherlands Antilles (Curacao, Bonaire, Saba, Sint Eustatius and Sint Maarten) and Aruba. The socio-economic position of these ‘colonial migrants’ is very diverse, but in general more favourable than that of those from the former labour recruitment countries (Van der Werfhorst and Van Tubergen 2007), not least because of their command of the Dutch language upon arrival (Vermeulen and Penninx 2000).

Although these four groups are the largest non-western migrant origin groups, the total migrant population of the Netherlands is much more diverse. Large migration flows followed the independence of Indonesia, the former Dutch colony of Dutch East Indies. Although this group is quite heterogeneous, the Indonesian community is generally considered well integrated and has a favourable socio-economic position. In addition, the majority of people with a migrant background in the Netherlands are from other EU countries (Van Wissen and Heering 2014; Van Mol and De Valk 2016). Over the past decade, migration from new EU-member states in Eastern Europe, and Poland in particular, has increased sharply (Gijsberts and Lubbers 2013). Since the focus of this paper is mainly on the methodological contribution of individualized neighbourhoods compared to administrative or statistical neighbourhoods, we restrict the analysis to a small number of comparable migrant origin groups, for which we can compare results to previously studied conventional segregation measures.

### Settlement Patterns of Migrant Origin Groups in the Netherlands

Prior evidence suggests that people of non-western migrant origin predominantly live in urban areas, and particularly in the western part of the country—the Randstad region—where the main economic and political centres are located (Musterd and Van Kempen 2009). International evidence shows that segregation also occurs in smaller towns (Malmberg et al. 2011) and even in rural areas (Lichter et al. 2012, 2015). Indeed, outside the Randstad region, strong concentrations are particularly found in regions with an economic profile (previously) dominated by manufacturing in the southern and eastern parts of the country. An example is the Turkish community in the eastern region of Twente, where previously the textile industry was an important source of labour (Das 2010). Also the Antillean community has some notable concentrations outside the Randstad area.

However, also within cities, people of non-western migrant origin appear to settle in specific neighbourhoods and their strongest concentrations often coincide with socio-economic inequalities: districts with relatively many low-income households and high welfare dependency (Hartog and Zorlu 2009). However, ethnic segregation does not only follow income and class patterns and related housing market constraints. Ethnic concentrations may also be the result of congregation: a deliberate choice for living among co-ethnics (Van Ham and Manley 2009; Musterd and Van Kempen 2009; Johnston et al. 2016). For recent arrivals in particular, initial location choices are often driven by the presence of co-ethnics and neighbourhood economic conditions (Zorlu and Mulder 2008). Historically grown migrant communities may reinforce concentrations of certain migrant origin groups in specific neighbourhoods, as was shown for the Netherlands (Zorlu and Latten 2009; Boschman and Van Ham 2015) and the UK (Van Ham and Clark 2009; Van Ham and Manley 2009). Subgroups within the non-western migrant origin population appear to live in different neighbourhoods. Earlier empirical studies found that there is some overlap in the residential patterns of the Turkish and Moroccan groups, but the Surinamese and Antilleans generally concentrate in other areas (Hartog and Zorlu 2009).

Neighbourhoods with a high concentration of migrants and their descendants can either be mixed, with immigrants of diverse origins, or specialized, with a large concentration of one particular group. The latter type of district is not common in The Dutch context, at least not at the level of administrative neighbourhoods. Nonetheless, there are examples of districts where the majority of the population is of non-western migrant origin. Still, the large majority of people with a migrant origin in the Netherlands (80%) live in neighbourhoods where those of the same country make up less than 10% of the total population (Hartog and Zorlu 2009). Although the concentration patterns of people of non-western migrant origin in the Netherlands are generally stable, there has been a gradual shift from inner-city districts towards post-war neighbourhoods since the 1990s (Bolt et al. 2002).

### Settlement of Migrant Origin Groups in the Amsterdam and Rotterdam Metropolitan Regions

**Fig. 1:**
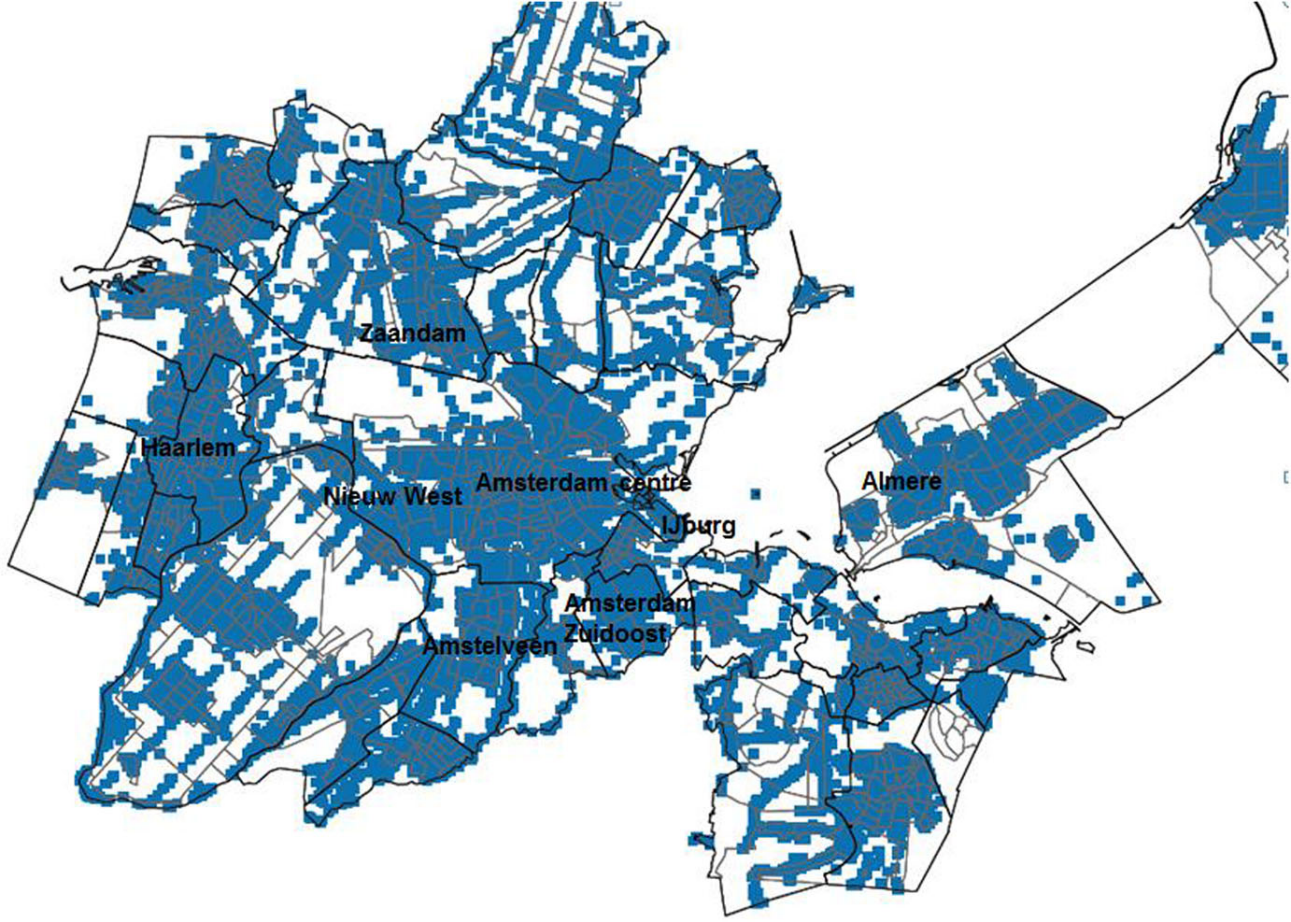
Amsterdam Metropolitan Area. *Source*: Statistics Netherlands 2011 and Kadaster 2011

This paper focuses on patterns of segregation in the entire country of the Netherlands, but we also zoom in on the two largest metropolitan regions: the Amsterdam Metropolitan Area and the Greater Rotterdam Area (*Stadsregio Rotterdam*). The city of Amsterdam had 810,935 inhabitants in 2011 and is the capital and main financial hub of the Netherlands (Fig. 1). The entire metropolitan area has approximately 2.3 million inhabitants. Amsterdam is a highly diverse city with approximately 180 nationalities. All of the four largest non-western migrant origin groups are well represented in the region and show the strongest concentrations on the western and south-eastern edges of the city of Amsterdam. The Moroccan origin group is the relatively largest minority in the city of Amsterdam (9%), closely followed by the Surinamese (8%). The Turkish origin group is somewhat smaller (5%), whereas people with a migrant background from the Netherlands Antilles and Aruba make up 1% of the total population. For the Surinamese origin group, the Amsterdam region has by far been the most important settlement region in absolute terms, since the independence of Surinam in 1975. Previous studies have shown that the Surinamese and Antillean origin groups predominantly live in the south-eastern borough of *Amsterdam Zuidoost*, a post-war district characterized by large high-rise estates. However, there has also been some suburbanization of the Surinamese origin group to the newly built area of *IJburg* and the suburb of Almere. The best-known concentrations of the Turkish and Moroccan origin groups are in the western part of the city—the borough of *Nieuw*-*West*—and to a lesser degree in the eastern and northern parts. The central neighbourhoods are characterized by increasing levels of gentrification, which has led to diminishing shares of the non-western migrant origin groups over the past two decades. There are relatively many migrants from European and other industrialized countries living in the central and more upmarket districts, however. Amsterdam is attractive for these groups mainly due to the presence of large multinational companies and two universities.

**Fig. 2:**
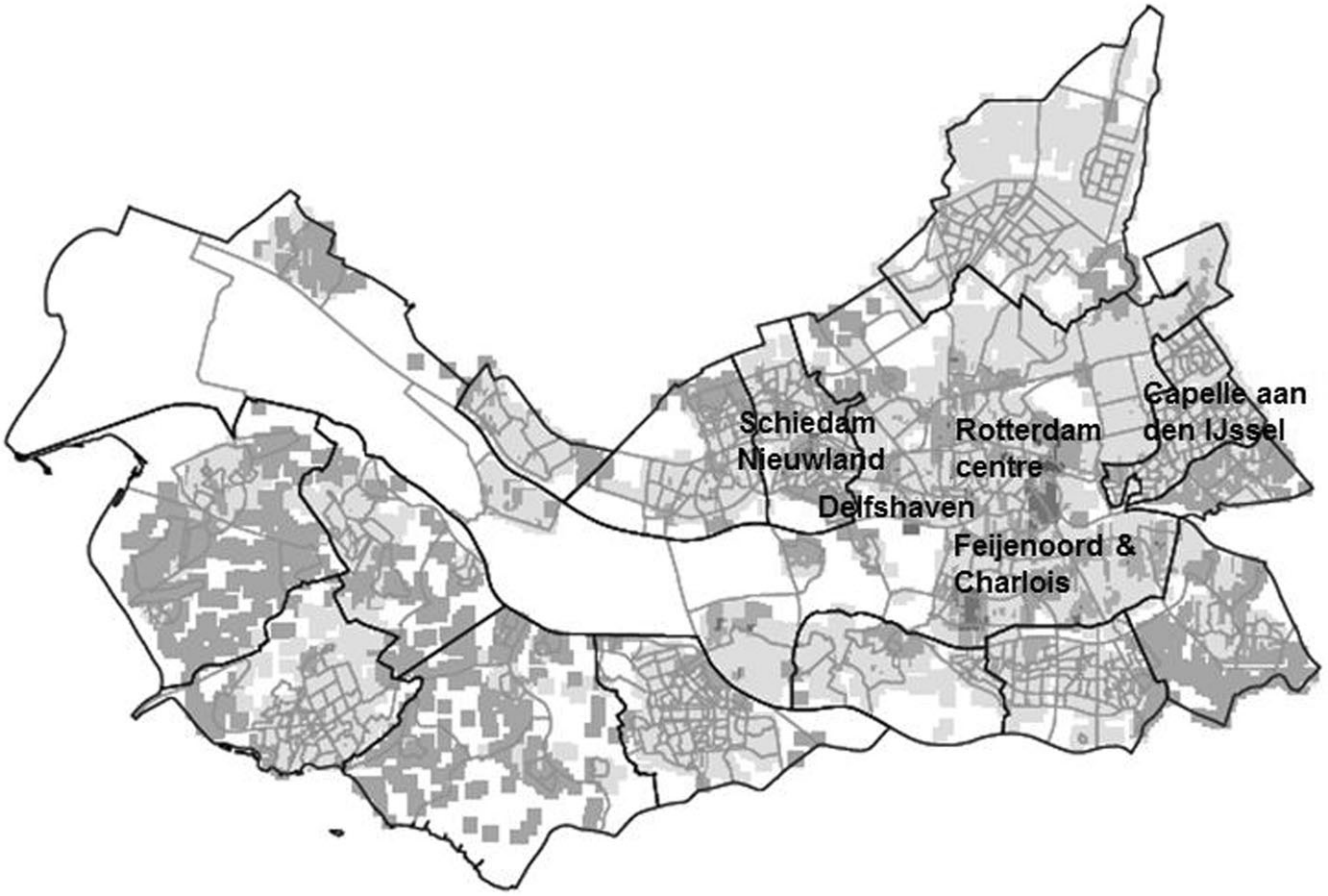
Greater Rotterdam Area. *Source*: Statistics Netherlands 2011 and Kadaster 2011

Rotterdam (618,355 inhabitants in 2011) is the second-largest city and the major port of the Netherlands. The entire Greater Rotterdam Area has approximately 1.2 Million inhabitants (Fig. 2). The city has a strong industrial past, which has attracted large numbers of labour migrants since the 1960s. The Turkish and Surinamese origin groups are the largest non-western minorities, both with around 8% of the total population. Compared to Amsterdam, the Moroccan origin group is somewhat smaller (7%), but the Antillean/Aruban community is much larger (4%). The largest concentrations of migrant origin groups in the Rotterdam region are the southern boroughs of *Feijenoord* and *Charlois*, the western borough of *Delfshaven* and the *Nieuwland* district in the adjacent city of Schiedam.

## Ethnic Segregation as a Multiscalar Phenomenon

Segregation studies often deal with the overrepresentation of migrant origin groups or low-income households in certain neighbourhoods (Massey and Denton 1988; Musterd and Ostendorf 2009; Nijkamp and Poot 2015). Segregation measurements are highly influenced by the spatial units for which they are measured, and using different units may lead to different results (Jones et al. 2015). For example, the ‘isolation index’, which measures the degree of exposure, increases with area size and is strongly dependent on general minority shares (Malmberg et al. 2011).

Most studies have analysed segregation for administrative or statistical districts with fixed boundaries. These areas are measured differently across countries, and there are also national and even regional variations in terms of their average population size. Some of these districts are relatively large entities with on average between 3000 and 6000 residents, such as census tracts in the USA (Galster et al. 2000; Lichter et al. 2012). In the Dutch context, the districts according to the Statistics Netherlands definition or postal code areas are most used (Hartog and Zorlu 2009; Musterd et al. 2012). Examples of smaller entities with fixed geographical borders are wards and output areas in the UK (Manley et al. 2012) and SAMS (Small Area Market Statistics) in Sweden (Musterd and Andersson 2005). These differences in population size between statistical units affect all quantitative analyses making use of geographical delineations and impede reliable cross-regional and international comparisons: the MAUP (Openshaw 1984; Malmberg et al. 2011, 2014).

Traditional measurements of segregation have often focused on one spatial scale. Fowler (2015) recently argued that segregation is continuous across different scale levels, and there is no single ‘correct’ scale for calculating measures of segregation. Which scale level is the most relevant for neighbourhood effects depends strongly on the specific topic under study, population density and also differs between age categories. For example, with respect to socialization, the local level may be the most relevant scale level for children, whereas larger spatial scales become more important in later stages of the life cycle, when activities and social networks generally exceed neighbourhood borders (Andersson and Musterd 2010). Single scalar measurements may ignore the broader context in which a neighbourhood is located. Within larger entities with low or moderate segregation levels, very strong concentrations may exist at smaller spatial scales, or vice versa. Focusing on only one spatial scale may overlook specific ethnic concentrations (Fowler 2015). Furthermore, fixed borders may lead to over- or underestimations of very specific concentrations that occur at the border of two administrative or statistical districts (Clark et al. 2015; Hedman et al. 2015).

In another recent study, Jones et al. (2015) argued that scale is important for understanding the causes and impact of segregation. In their study, the strongest concentrations of most (though not all) ethnic groups were found at both the largest and the smallest scales: they are clustered into boroughs and in several small areas within them. Clark et al. (2015) argued that multiscalar measures help to understand neighbourhood dynamics better, since they enable a link between actual changing patterns of segregation and experiences of changing population compositions in individuals’ residential locations. They suggested that rather than focusing on units with fixed borders, segregation should be studied at different spatial scales and for units with fixed population sizes in which an individual is the centroid.

The increased availability of geocoded individual data offers opportunities for solving above-mentioned boundary and scale issues, by constructing scalable individualized neighbourhoods. These districts are ‘egocentric’: the exact residential location of an individual is taken as the centroid, from where a buffer is constructed that consists of a predefined distance radius (Reardon et al. 2008) or a *k*-number of nearest neighbours (*k*-levels) (Andersson and Malmberg 2015; Östh et al. 2014). The resulting sample of individuals is then used to compute aggregate statistics, such as the share of people belonging to a certain migrant group. As long as geocoded information is available, individualized neighbourhoods can be calculated in the same way for different countries (Clark et al. 2015). Since the number of nearest neighbours within the buffer can vary, individualized neighbourhoods of different sizes seen from the same location can be studied enabling analysing residential segregation from a multiscalar perspective.

Previous research showed stronger neighbourhood effects for the smallest individualized neighbourhoods. Using individualized neighbourhoods based on nearest neighbours, Andersson and Malmberg (2015) find that the effects of role models, norms and peer effects on educational outcomes are three times greater in the smallest individualized neighbourhoods than in administrative or statistical neighbourhoods in Sweden (SAMS). MacAllister et al. (2001) found similar effects in their study on voting behaviour in the UK: significant differences in voting behaviour within social classes were detected at the smallest scale levels, according to the socio-economic status of their individualized neighbourhood. Also, Chaix et al. (2005) found much stronger relationships between contextual deprivation and the prevalence of disorders in individualized areas of smaller size than in administrative neighbourhoods. The immediate environment of individual residential locations presumably has a larger impact on personal outcomes and household decisions than the population composition of administrative or statistical districts (Clark et al. 2015).

## Data and Methodology

### Data

Data for this study come from the System of Social Statistical Datasets (SSD) of Statistics Netherlands. This is a system of linked statistical registers and surveys, starting in 1996, which cover a broad range of demographic and socio-economic subjects in which data from various sources, such as municipal population registers, tax offices, labour offices and public education institutes, are combined (Bakker et al. 2014).

Here we use the most recent available data at the start of the project covering all persons who were included in the municipal population registers at the end of 2011 (total population: 16,731,357). We distinguish persons of migrant origin from the native Dutch population by the person’s own country of birth or the country of birth of one of the parents.^1^

### Measuring Administrative and Individualized Neighbourhoods

For each person, the SSD database provides information on addresses, although the actual addresses are replaced by a unique numeric code for privacy reasons. For each address, we know in which administrative entity it is located. Municipalities are the largest entities and range in size from 900 inhabitants to over 800,000 residents (Amsterdam). These municipalities are subdivided into a small number of boroughs, which in turn are comprised of several neighbourhoods. The borders of these administrative neighbourhoods are drawn by the municipality in which they are located. For each administrative unit in the Netherlands, we know the total population and also the number of persons belonging to a specific minority group. The size of these areas differs strongly across the country, largely depending on population density. The average size of a neighbourhood in the Netherlands is 1442 inhabitants, but in the large cities these areas are generally larger: 8228 on average in Amsterdam and 7164 in Rotterdam. The average population size of boroughs is 6523 in the Netherlands as a whole, but, respectively, 98,740 and 32,427 in Amsterdam and Rotterdam.

In addition, each address is linked to a unique combination of geocoordinates: a x-coordinate indicating the location on a line running from east to west and a y-coordinate indicating the location on a line running from north to south. The coordinates for the Netherlands are based on the *‘Rijksdriehoeksstelsel’* (RD), which is compatible with the European Terrestrial Reference System 1989 (ETRS89) and maintained by the national cadastre.

These coordinates are the input for the measurement of individualized neighbourhoods. We construct individualized neighbourhoods based on a *k*-number of nearest neighbours (*k*-levels). In this paper, eleven *k*-levels are taken into account, with small (50, 100, 200 and 400), medium-sized (800, 1600, 3200 and 6400) and large population counts (12,800, 25,600 and 51,200 nearest neighbours). All individual addresses are grouped into grids of 100 by 100 ms. From each 100 by 100 m grid, the Swedish geographical information system *EquiPop* (see Östh 2014 for a detailed description) can find nearest neighbours in the adjacent grids, based on the geocoded information.

**Fig. 3:**
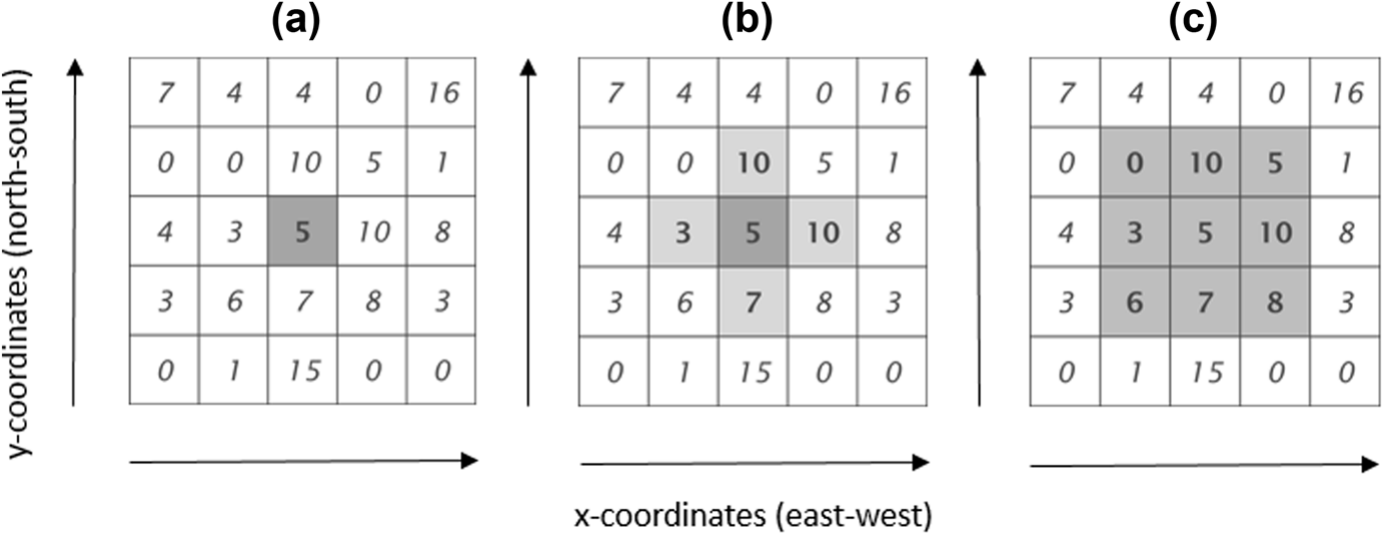
Constructing an individualized neighbourhood of 50 nearest neighbours (*k* = 50), using the EquiPop software

Figure 3 illustrates how EquiPop constructs buffers with a *k*-number of nearest neighbours from each grid. In this example, we end with an individualized neighbourhood of *k* = 50, with 54 residents. This sample of individuals is used to compute aggregate statistics, including the ratios of specific migrant origins.^2^

### Methodology

The empirical section of this study consists of two parts. First, we construct neighbourhood typologies based on the ratios, or relative shares, of different population groups in the total population of each spatial unit. We take the ratios of the four largest non-western migrant origin groups in the Netherlands—Moroccans, Turks, Surinamese and Antilleans—and of the native Dutch population (Table 1).

**Table 1:** Description of indicators used in the factor analyses

Indicator
A Ratio of native Dutch within total population: both parents were born in the Netherlands
B Ratio of Moroccan origin within total population: person or at least one parent was born in Morocco
C Ratio of Turkish origin within total population: person or at least one parent was born in Turkey
D Ratio of Surinamese origin within total population: person or at least one parent was born in Surinam
E Ratio of Antillean origin within total population: person or at least one parent was born in the Netherlands Antilles

For each indicator, we calculate ratios for administrative units at three spatial scales (neighbourhoods, boroughs and municipalities) and for individualized neighbourhoods at all eleven *k*-levels. This means that for the administrative neighbourhoods, we have fifteen indicators: the ratios of each indicator for each administrative neighbourhood, borough and municipality. For the individualized neighbourhoods, we have 55 indicators for each unique pair of coordinates: the ratios of each indicator for individualized neighbourhoods at 11 scales. For example, indicator A *k* = 50, indicator A *k* = 400, […], indicator A *k* = 51,200, and this will be repeated for each indicator.

Second, we apply factor analysis to synthesize this range of indicators into a smaller number of components. Since there is a strong correlation between migrant origin group ratios measured across different spatial scales, the measurements of the same indicator at each scale will likely end up in the same factors. Still, variable A at *k* = 50 may have a different factor loading than variable A at *k* = 51,200. The factor loadings indicate which spatial scales are captured most strongly by each factor (see Malmberg et al. 2014 for a similar application). The factor analyses are based on correlations and only principal components with an eigenvalue higher than 1 were rotated, using the varimax rotation. Based on the factor scores, we identify different neighbourhood types, where high scores on one indicator correlate with high or low scores on others. We conduct factor analyses based on administrative neighbourhoods, boroughs and municipalities, and then repeat the analysis for individualized neighbourhoods. The aim is to analyse whether the multiscalar individualized neighbourhood approach results in a different neighbourhood typology based on ethnic heterogeneity, compared to the fixed administrative units. The geographical dispersion of factor scores across the Netherlands and across the two largest metropolitan areas (Amsterdam and Rotterdam) is illustrated with maps, using the software Q-GIS.

Third, we calculate scores on the ‘isolation index’ for all four non-western origin groups for both administrative neighbourhoods and scalable individualized neighbourhoods. We here zoom in on the largest metropolitan region of the Netherlands, the Amsterdam Metropolitan Area. The isolation index is the most common measure of ‘exposure’: the degree of potential exposure of individuals to members of their own group (Massey and Denton 1988; Nijkamp and Poot 2015). This index is measured as follows:

$\sum\nolimits_{i = 0}^{n} {\left[ {\left( {\frac{{x_{i} }}{X}} \right)\left( {\frac{{x_{i} }}{{t_{i} }}} \right)} \right]} ,$ where $x_{i}$ is the minority population of area i, X is the total minority population and $t_{i}$ is the total population of area i (Iceland et al. 2000).

The isolation index is measured for a city or—in this case—a metropolitan region and is the sum of all scores for all its individual districts. The index ranges from 1, where a minority member will likely only encounter co-ethnics within a given area to 0, where this chance is absent (Malmberg et al. 2011).

For administrative districts (in which the sum of all districts $\left( {t_{i} } \right)$ equals the total population (X)), the above-mentioned formula is useful, this is not the case for non-static individualized neighbourhoods. In individualized districts, the same people may be counted as nearest neighbours several times, which means that the sum of all individualized neighbourhoods does not equal the total population of a metropolitan area. Therefore, a weighted average for the minority population in each grid cell is used, leading to the following formula:

$${\text{Spatial}}\;{\text{Isolation}}_{\text{k}} = \sum\limits_{i = 1}^{n} {\frac{{\left( {x_{i} *\frac{{x_{i,k} }}{k}} \right)}}{{\left( {x_{i} } \right)}}}$$

In this formula, $x_{i}$ stands for the size of the local minority population in each grid cell, whereas $\frac{{x_{i,k} }}{k}$ represents the share of this minority group within the total population of each *k*-level. We subsequently compare the resulting isolation index scores for administrative and individualized neighbourhoods in order to see whether and how the new approach leads to a different estimation than the traditional measurement.

## Neighbourhood Typologies Based on Administrative and Individualized Neighbourhoods

**Fig. 4:**
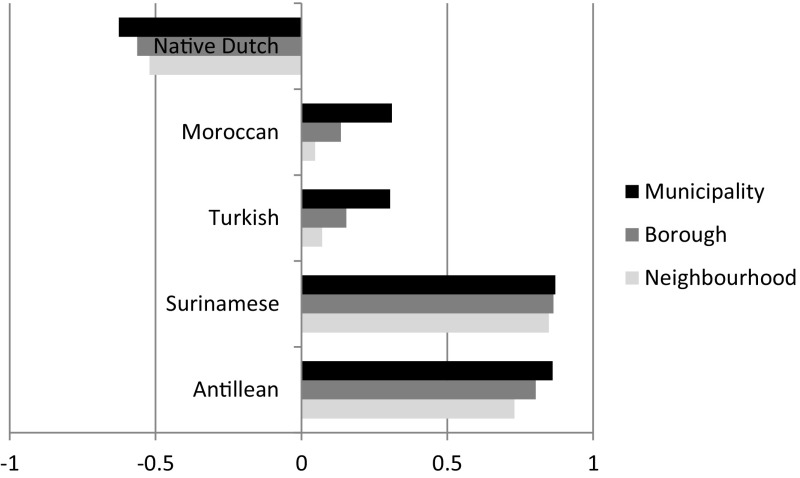
Factor loadings for Administrative Factor 1: ‘Surinamese and Antillean clusters’. *Source*: Statistics Netherlands, 2011

We first conducted a factor analysis based on three administrative scale levels, according to the Statistics Netherlands definition: neighbourhoods, boroughs and municipalities. This factor analysis resulted in three factors (see “Appendix” for detailed results). Factor 1, with an explained variance of 35.4%, can be interpreted as ‘Surinamese or Antillean clusters’ (Fig. 4). The factor loadings for the Surinamese origin group remain relatively stable around 0.8 across the three spatial scales, whereas the loadings for the Antillean origin group increase slightly from 0.73 at the neighbourhood level to 0.86 at the municipality level. Within the Factor 1 districts, the Turkish and Moroccan origin groups are hardly represented at the neighbourhood level but have moderate concentrations at the municipality level: the factor loadings are around 0.31. This suggests that the Surinamese and Antillean origin groups live in similar districts, whereas the Turkish and Moroccan origin groups live in different districts but often in the same municipalities.

**Fig. 5:**
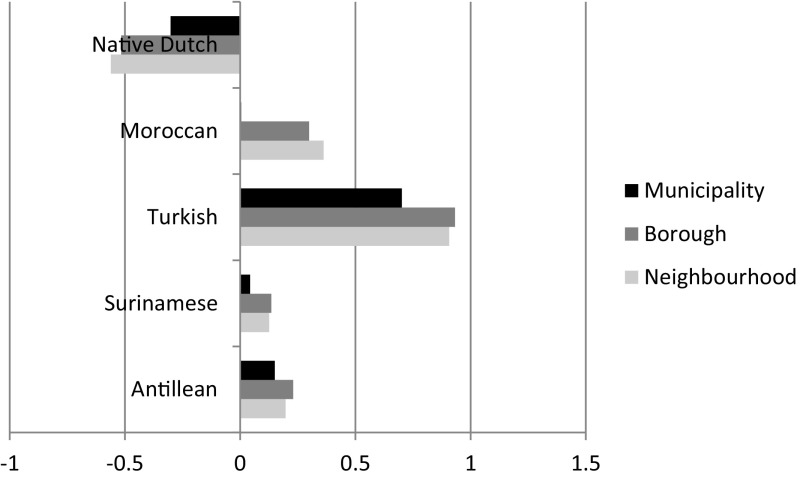
Factor loadings for Administrative Factor 2—‘Turkish clusters’. *Source*: Statistics Netherlands 2011

The second factor, with an explained variance of 21.5%, represents ‘Turkish clusters’. Here, people of Turkish origin are strongly represented at all, but particularly at the smaller and medium-sized, scales (Fig. 5). The factor loading for the Turkish origin group is 0.90 at the neighbourhood level and slightly increases to 0.93 at the borough level. At the municipality level, the factor loading has dropped to 0.70. The Moroccan origin group has moderate concentrations in Factor 2 at the neighbourhood level (factor loading 0.63), but the factor loading decreases with each increase in scale: 0.30 at the borough level and 0 at the municipality level. The Antillean and Surinamese origin groups have only modest concentrations in Factor 2, with the highest factor loadings at the borough level (0.23 and 0.14, respectively). The interpretation of this is that the Turkish origin group is segregated at all three spatial scales. They live in specific municipalities, but also in different boroughs and neighbourhoods within them. They also live in different districts than the other three non-western origin groups and the natives, although sometimes there are overlaps with the concentration areas of the Moroccans.

**Fig. 6:**
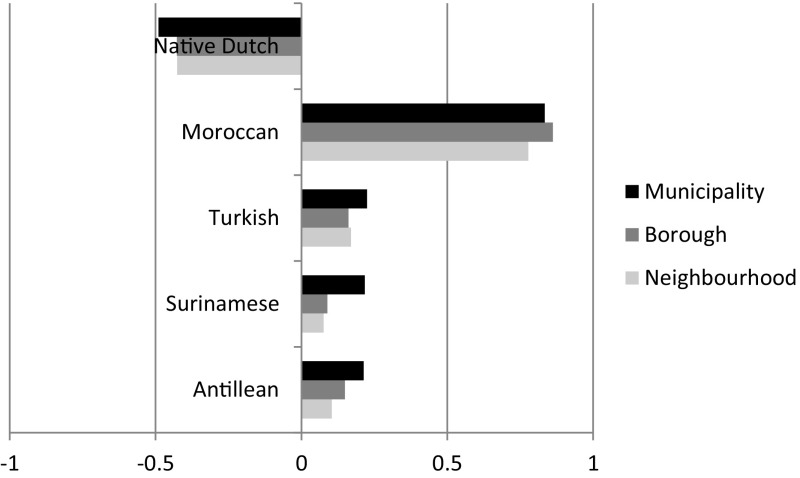
Factor loadings for Administrative Factor 3—‘Moroccan clusters’. *Source*: Statistics Netherlands 2011

Factor 3 (explained variance: 19.3%) depicts ‘Moroccan clusters’ (Fig. 6). The factor loading for the Moroccan origin group remains relatively stable around 0.8 across all three scale levels. All other three origin groups have only weak concentrations in the Factor 3 districts; only at the municipality level their factor loadings are higher than 0.20. This suggests that the Moroccan origin group lives in specific municipalities and also within them in particular boroughs and neighbourhoods.

### Neighbourhood Typologies Based on Individualized Neighbourhoods

The second factor analysis included individualized neighbourhoods of eleven different scales (see “Appendix” for detailed results). A first notable finding is that the approach with individualized neighbourhoods results in a larger number of factors with an eigenvalue higher than 1: seven in total, with a total explained variance of 89.3%. Below, we will describe four of these factors according to their factor loadings and the geographical dispersion of factor scores. The factor loadings indicate at which scale levels the factors have their main effects. Some factors influence the immediate surroundings (smaller *k*-levels), other factors influence population compositions of larger neighbourhood units (medium-sized and larger *k*-levels), while others have effects across all eleven spatial scales.

**Fig. 7:**
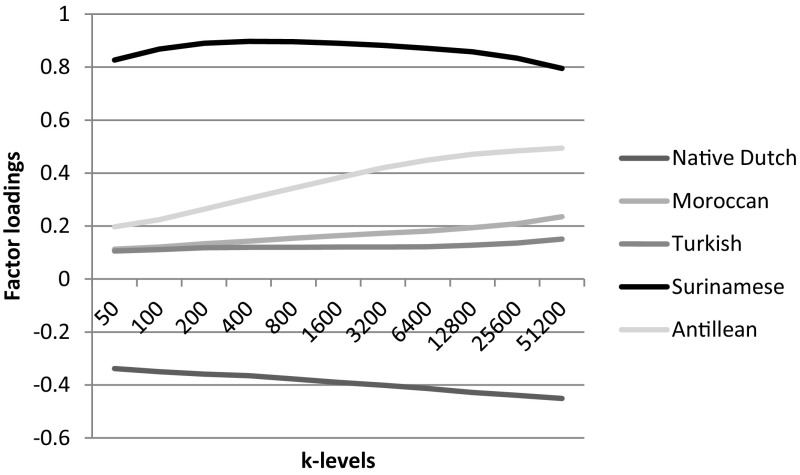
Factor loadings in the Netherlands for Individualized Factor 1—‘Surinamese clusters’. *Source*: Statistics Netherlands 2011

**Fig. 8:**
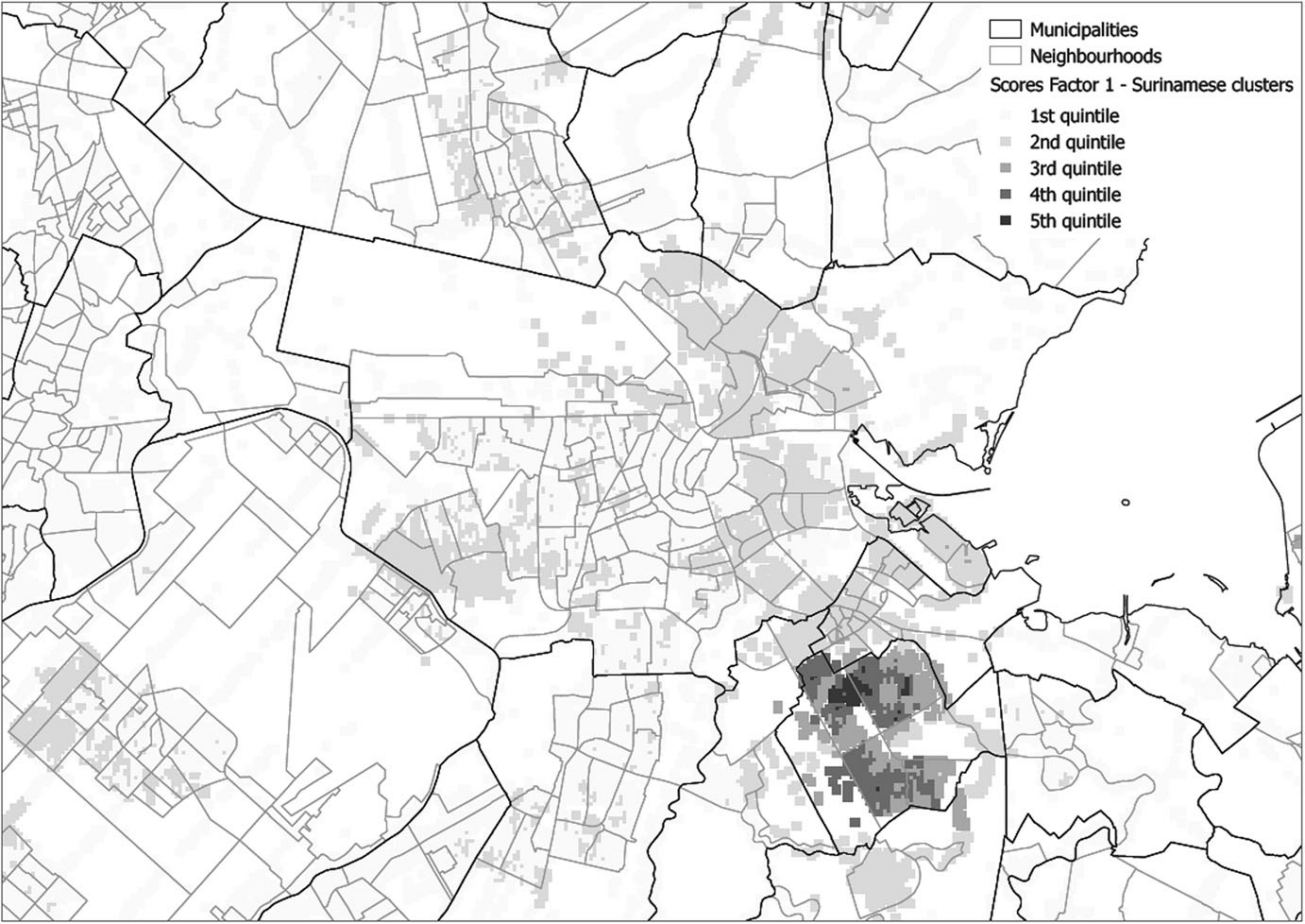
Geographical dispersion of factor scores in Amsterdam for Individualized Factor 1—‘Surinamese clusters’. *Source*: Statistics Netherlands 2011 and Kadaster 2011

The first factor can be identified as ‘Surinamese clusters’ (explained variance 21.8%). In areas with a high score on this factor, people of Surinamese origin are the main minority group. Figure 7 shows that the concentration of the Surinamese origin group in this factor is relatively constant across spatial scales. At all eleven *k*-levels, the factor loading for the Surinamese origin group lies between 0.8 and 0.9. Their relatively stable concentrations across all scales can be explained by the fact that their main cluster is one specific, large borough of Amsterdam (*Zuidoost*, see Fig. 8). Even though there are differences within Zuidoost, the borough as a whole has much larger shares of the Surinamese origin group compared to other parts of Amsterdam and, especially, areas outside Amsterdam. The Surinamese origin group lives in only a limited number of locations within the Netherlands. The main concentration area is the Amsterdam region, including the suburb of Almere, but to a lesser extent also the Hague and Rotterdam have notable concentrations.

At the smallest spatial scales all other groups are not strongly represented in Factor 1. At the medium-sized and larger spatial scales, we do find relatively strong concentrations of the Antillean origin group as well. At *k* = 50, the Antillean origin group has a factor loading of 0.2, but this increases to 0.5 at *k* = 51,200. The Moroccan and Turkish origin groups have low factor loadings, which largely remain stable across spatial scales. This suggests that persons of Surinamese origin generally live geographically separated from the Turkish and Moroccan origin groups, but the Antillean origin group has some concentrations in the Surinamese clusters.

**Fig. 9:**
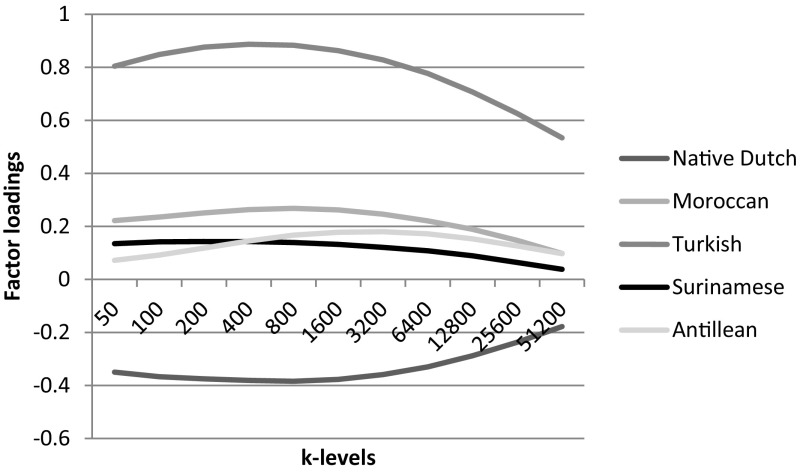
Factor loadings of factor scores in the Netherlands for Individualized Factor 2—‘Turkish clusters’. *Source*: Statistics Netherlands 2011

**Fig. 10:**
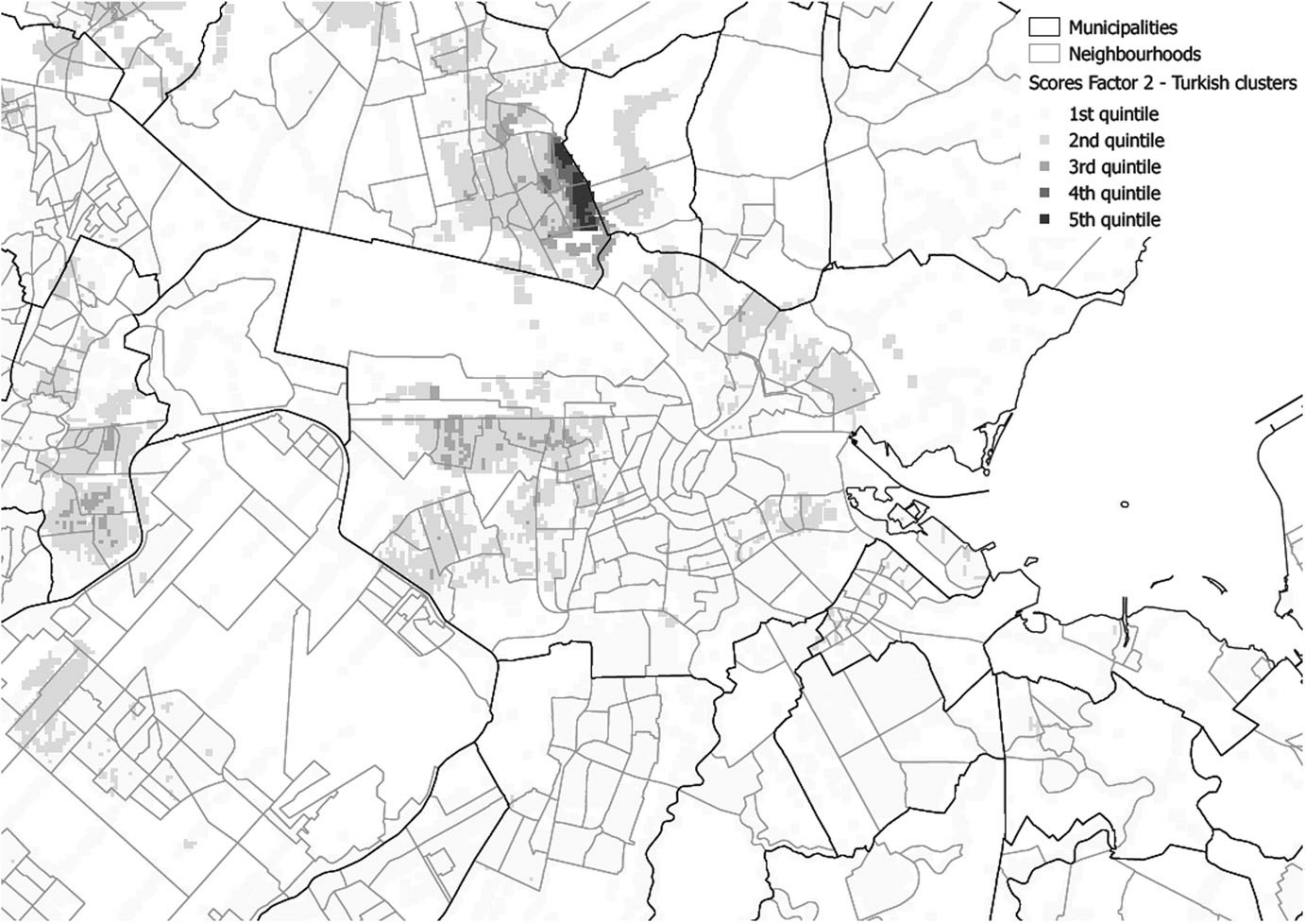
Geographical dispersion of factor scores in Amsterdam for Individualized Factor 2—‘Turkish clusters’. *Source*: Statistics Netherlands 2011 and Kadaster 2011

**Fig. 11:**
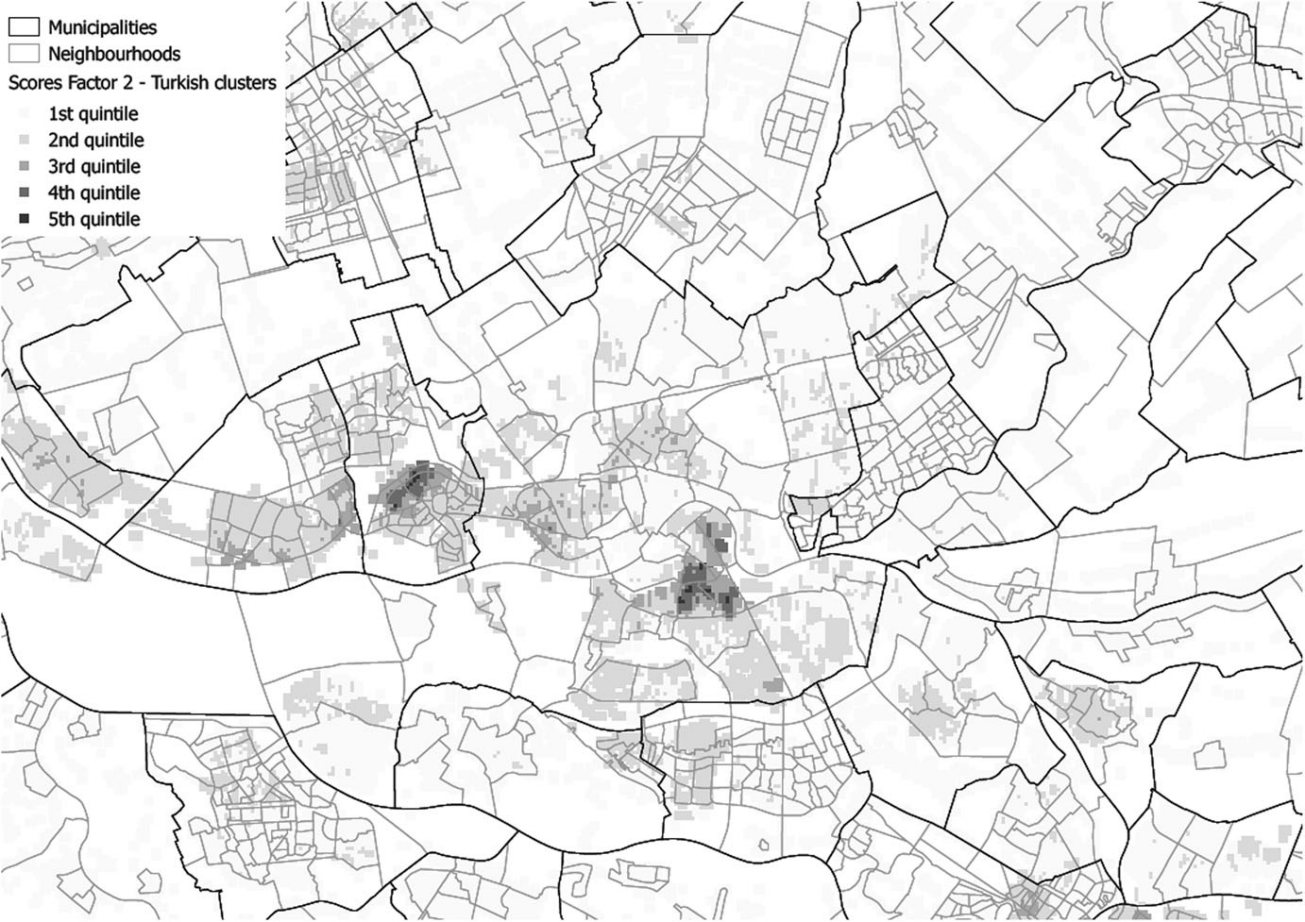
Geographical dispersion of factor scores in Rotterdam for Individualized Factor 2—‘Turkish clusters’. *Source*: Statistics Netherlands 2011 and Kadaster 2011

Individualized Factor 2 can be characterized as ‘Turkish clusters’ (explained variance 16.5%). Within this factor, persons of Turkish origin are the dominant origin group, but their concentrations differ across spatial scales (Fig. 9). At *k* = 50, the Turkish origin group has a factor loading of 0.8, which slightly increases up to 0.88 at *k* = 800. For scale levels beyond *k* = 800, the factor loadings for the Turkish origin group start decreasing: at *k* = 51,200 the factor loading has fallen to 0.5. This means that persons of Turkish origin are strongly segregated at smaller spatial scales, but moderately segregated at medium-sized and larger scales. The main concentration areas of the Turkish origin group are in specific, generally larger, municipalities in the western part of the country (Randstad), but also in (former) industrial towns in the south and east there are a few notable concentrations. Within these municipalities, the ‘Turkish clusters’ are restricted to a limited number of administrative neighbourhoods and even to specific smaller parts within them (see Figs. 10 and 11).

The ‘Turkish clusters’ are generally found in districts where there are no strong concentrations of other origin groups. For all other three non-western origin groups, the factor loadings are below 0.3, however, and remain relatively stable across the eleven spatial scales. Still, the Moroccan origin group has a slightly stronger presence in the ‘Turkish clusters’ than the Surinamese and Antillean origin groups

**Fig. 12:**
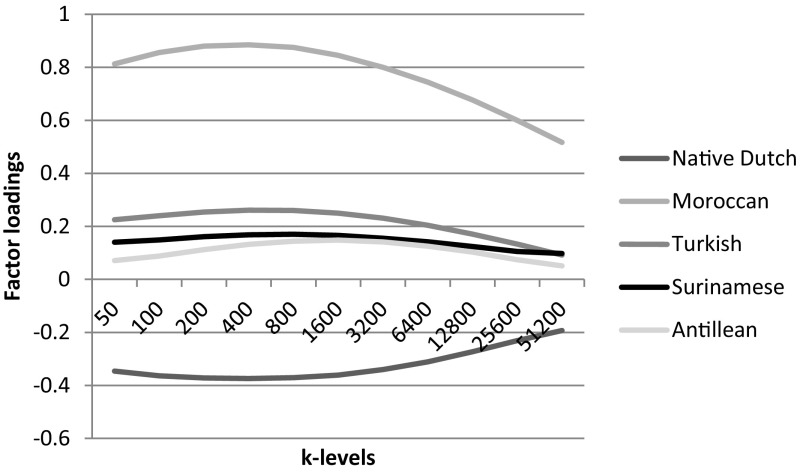
Factor loadings of factor scores in the Netherlands for Individualized Factor 3—‘Moroccan clusters’. *Source*: Statistics Netherlands 2011

Figure 12 shows the factor loadings for Individualized Factor 3: ‘Moroccan clusters’ (explained variance 16%), in which the Moroccan origin group is the dominant migrant origin group. The Moroccan clusters closely resemble the Turkish clusters regarding concentrations across different scales. Factor loadings for the Moroccan origin group are particularly high at smaller spatial scales. At *k* = 50, the factor loading is 0.81 and the scores increase slightly up to 0.89 at *k* = 400, and start decreasing afterwards: at *k* = 51,200 the factor loading has fallen to 0.51. The Turkish origin group has a somewhat stronger presence than the Surinamese and Antillean origin groups. All three origin groups have factor loadings below 0.26, with highest loadings around *k* = 400, and compared to the Moroccan origin group, the differences across spatial scales are relatively small.

**Fig. 13:**
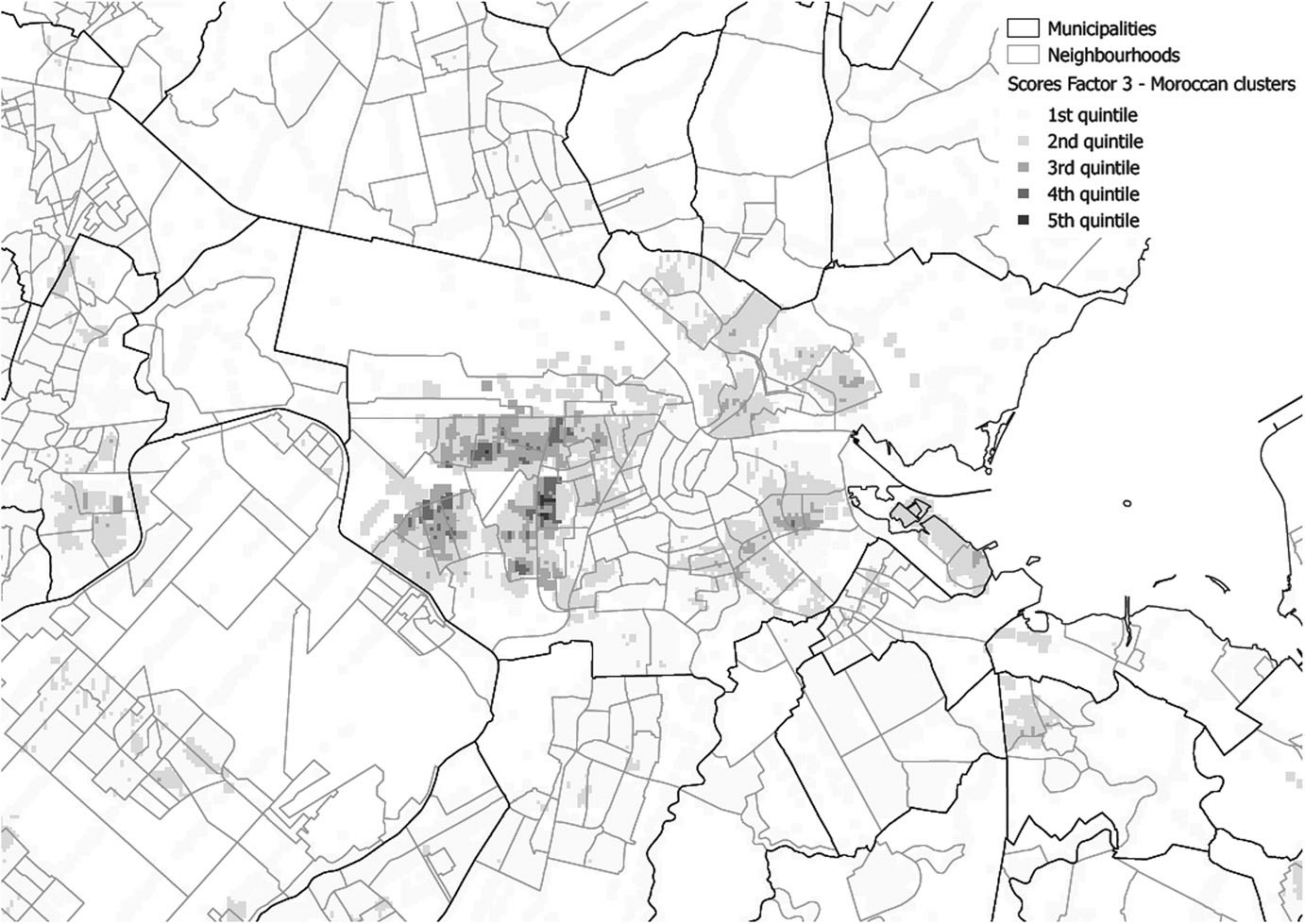
Geographical concentrations in Amsterdam for Factor 3—‘Moroccan clusters’. *Source*: Statistics Netherlands 2011 and Kadaster 2011

**Fig. 14:**
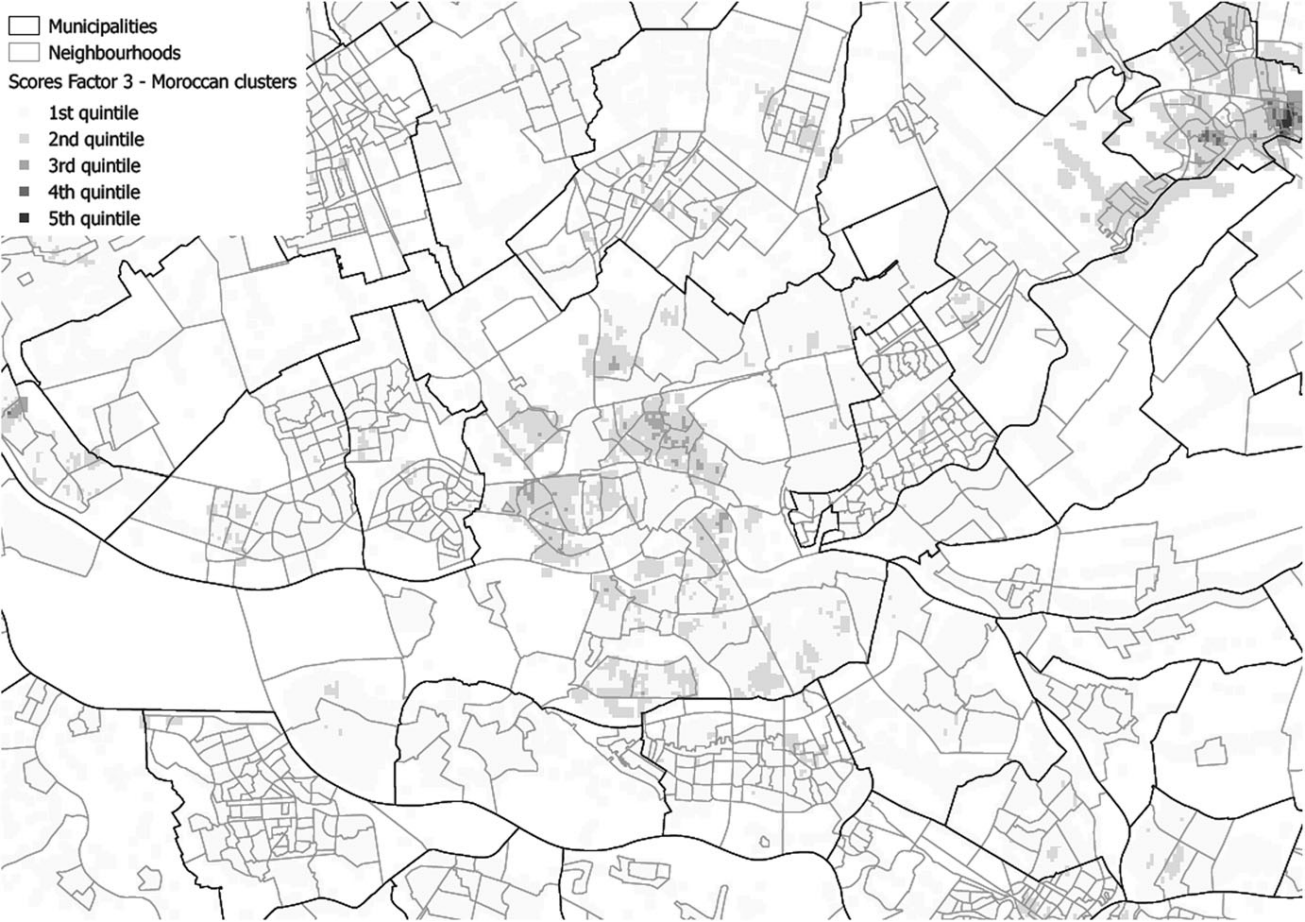
Geographical concentrations in Rotterdam for Factor 3—‘Moroccan clusters’. *Source*: Statistics Netherlands 2011 and Kadaster 2011

Despite the similarities in their concentration levels across spatial scales, we find that the Turkish and Moroccan clusters are situated in different districts. They are to a large degree found in different cities, but also within the Amsterdam and Rotterdam regions their clusters are found in different administrative districts and even in different parts of the same district (see Figs. 13 and 14). Also, the Moroccan origin group’s concentrations are largely restricted to a small number of large and medium-sized cities in the Randstad region (e.g. Amsterdam, Utrecht and Gouda), in contrast to the Turkish origin group which also has strong concentrations in smaller towns and in other parts of the country.

**Fig. 15:**
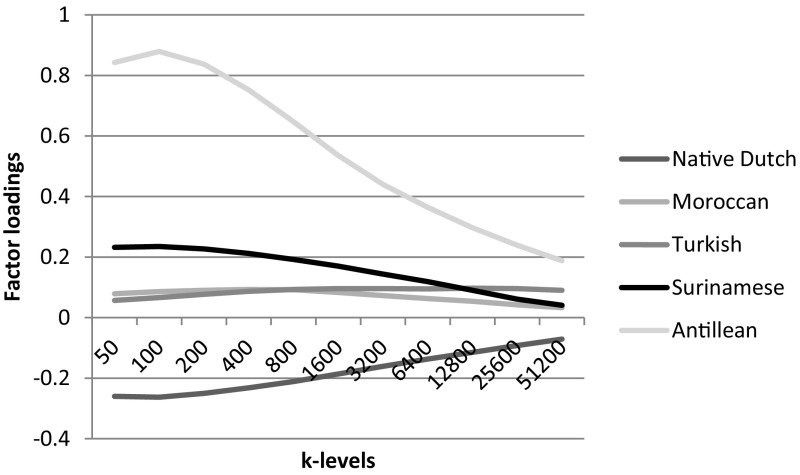
Factor loadings of factor scores in the Netherlands for Individualized Factor 6—‘Antillean clusters at small scales’. *Source*: Statistics Netherlands 2011

**Fig. 16:**
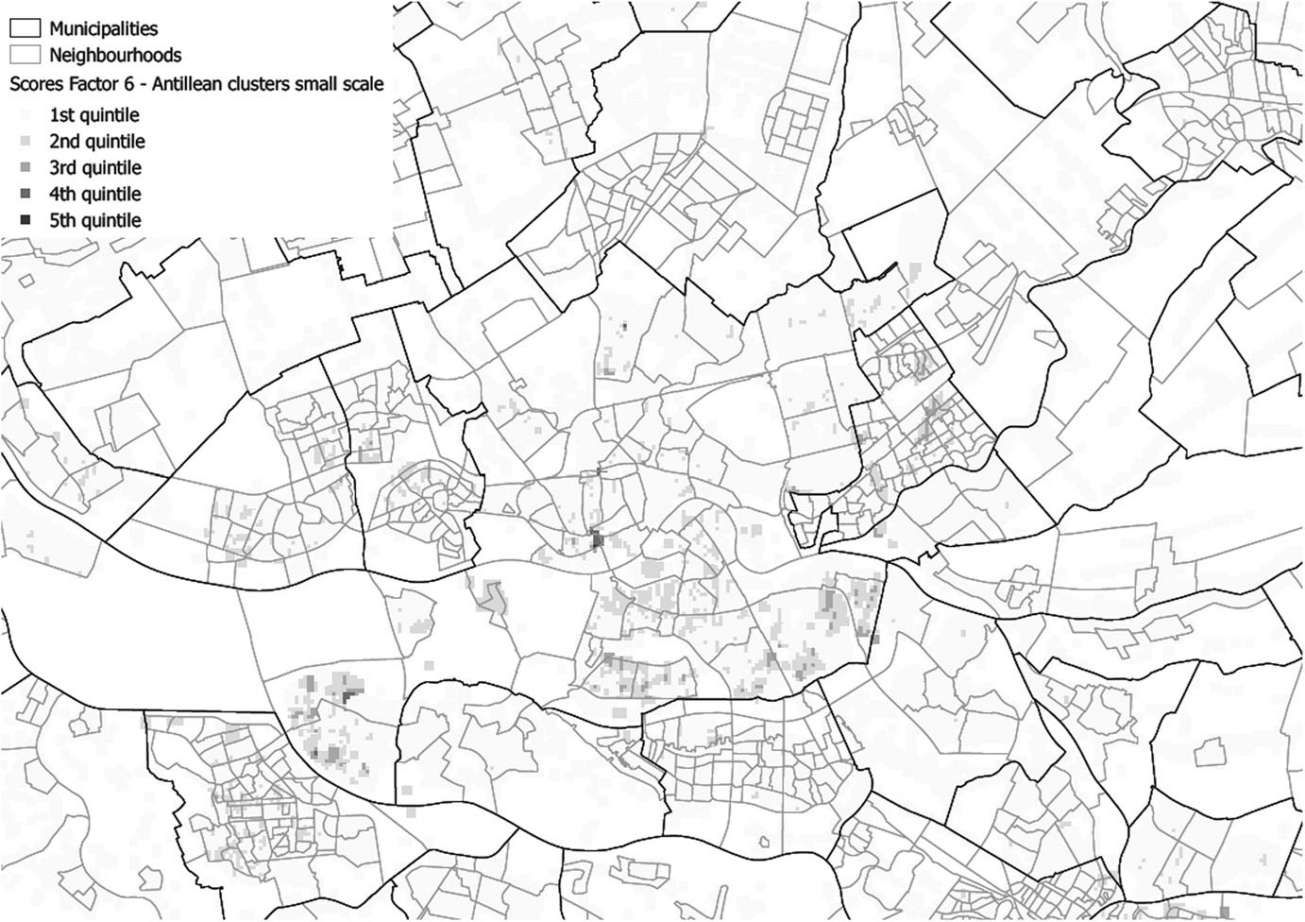
Geographical concentrations in Rotterdam for Individualized Factor 6—‘Antillean clusters at small scales’. *Source*: Statistics Netherlands 2011 and Kadaster 2011

Individualized Factor 6 (variance 8.8%) can be interpreted as ‘Antillean clusters at small scales’ (Fig. 15). Persons with a migrant background from the former Netherlands Antilles and Aruba are the dominant origin group in this factor, and their concentration patterns are a clear example of micro-level segregation. The factor loadings for the Antillean origin group are very high at the smallest spatial scales, but already beyond *k* = 100, a decrease can be observed, which becomes even stronger after *k* = 200. At the largest scales, the factor loadings are hardly higher than for the other origin groups. Figure 16 shows that within the Greater Rotterdam Area, the Antillean clusters are indeed localized in small sections of specific districts.

**Fig. 17:**
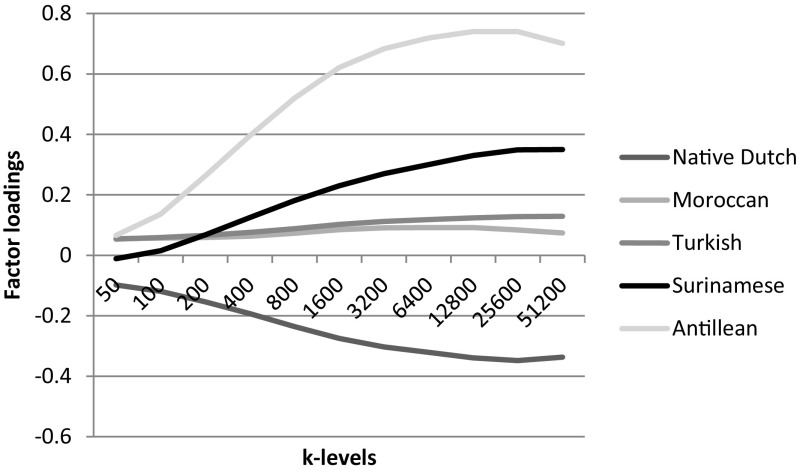
Factor loadings of factor scores in the Netherlands for Individualized Factor 4—‘Antillean clusters at larger scales’. *Source*: Statistics Netherlands 2011

Interestingly, we also found another factor for the Antillean origin group, which can be interpreted as ‘Antillean clusters at larger scales’ (explained variance 9.1%). The curve showing factor loadings across spatial scales (Fig. 17) is in fact the reverse of Factor 6: there are strong concentrations of the Antillean origin group at the largest spatial scales but hardly at the smallest. We can conclude from this that the Antillean origin group has strong concentrations at both small and large spatial scales. This can be explained by looking at the geographical dispersion of this origin group in the Netherlands: they live in only a small number of large and medium-sized cities, and also within these cities, their concentration patterns are highly localized. Additional analyses showed that the areas with high scores on Factor 6 are the districts where the Antillean origin group is actually strongly concentrated. The areas with high scores on the reversed factor are places within the same municipalities where the group is not strongly concentrated but which are still relatively near the districts where they do live.

Of the final two factors, one represented low concentrations of all four non-western origin groups at the smallest spatial scales, but high factor loadings for especially the Turks and Moroccans at larger spatial scales (9% explained variance). These areas turned out to be non-diverse enclaves in otherwise highly diverse administrative neighbourhoods and boroughs. The other factor (7.99% explained variance) had high factor loadings for the native Dutch and low for all four non-western origin groups, at all scales.

## Segregation Patterns and Levels Compared Across Administrative and Individualized Neighbourhoods: The Case of Amsterdam

In the next step, we calculated the Isolation index for all four non-western migrant origin groups, using both the conventional administrative neighbourhoods and individualized neighbourhoods. In this part, we just focus on the Amsterdam Metropolitan Area as a case study.

**Fig. 18:**
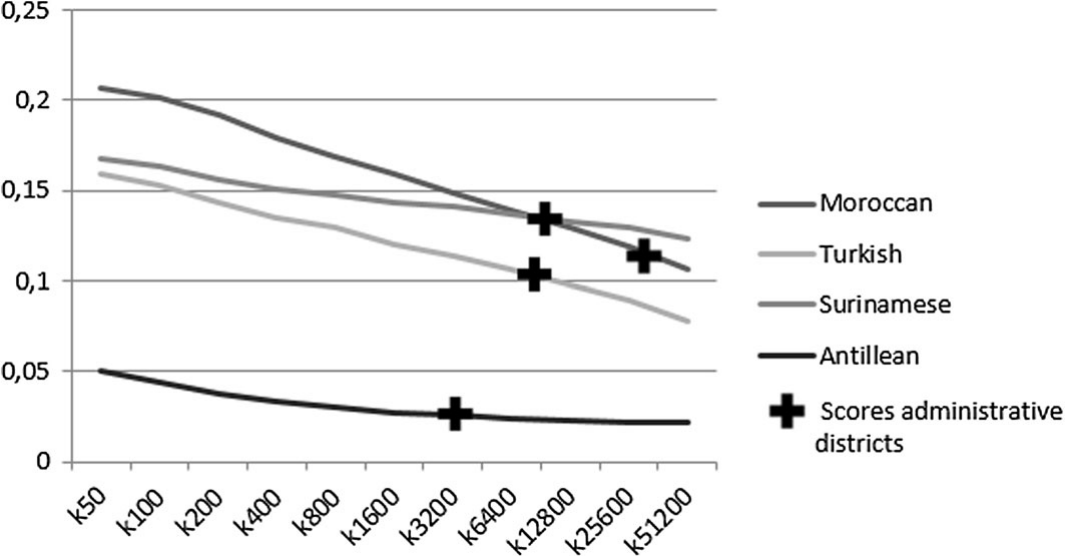
Isolation index scores of the Moroccan, Turkish, Surinamese and Antillean origin groups in the Amsterdam Metropolitan Area, based on individualized and administrative neighbourhoods. *Source*: Statistics Netherlands 2011

The isolation index score for the Moroccan origin group in the Amsterdam Metropolitan Area is 0.120 based on the sum of all administrative districts (Fig. 18). This means that the chance that someone of Moroccan origin will encounter a co-ethnic during a walk through the region is 12 per cent. However, when calculating the isolation index based on the sums of eleven scalable individualized neighbourhoods, we see that this chance is higher than 0.12 at all scales up to *k* = 12,800. At *k* = 25,600, the index scores are almost equal. The scores decrease from 0.21 at *k* = 50 to 0.11 at *k* = 51,200. For the Turkish origin group, we find that the isolation index score for administrative neighbourhoods equals the score for *k* = 6400 (0.11). For all smaller *k*-levels, the index scores are higher. The scores decrease quite rapidly, ranging from 0.16 at *k* = 50 to 0.08 at *k* = 51,200. The scores for the Surinamese origin group remain relatively stable, compared to the other groups; they range from 0.17 at *k* = 50 to 0.12 at *k* = 51,200. Using all *k*-levels smaller than *k* = 12,800 results in isolation index scores that are higher than the score based on administrative neighbourhoods (0.13). The most pronounced differences across spatial scales are found for the Antilleans, although they generally have low isolation index scores in the Amsterdam Metropolitan Area. The highest score is 0.05 at *k* = 50, which decreases to 0.027 at *k* = 1600 and remains relatively stable for the larger *k*-levels. The score for administrative neighbourhoods (0.025) ends up between the scores for *k* = 3200 and *k* = 6400.

The different scores on the isolation index show that chances of exposure are in fact highly scale-dependent and also differ strongly for each origin group under study. The results for individualized neighbourhoods result in different estimations of segregation across scale levels. Focusing only on administrative districts would underestimate the higher chances of exposure at the micro-scale level, while overestimating the lower chances at larger scale levels.

## Conclusions and Discussion

The main aim of this paper was to study to what extent using scalable individualized neighbourhoods leads to a more nuanced picture of segregation compared to using traditional administrative or statistical units. We first constructed neighbourhood typologies and second compared segregation index scores based on both types of measurement.

The results of our study underline the relevance of studying ethnic segregation at various spatial levels simultaneously (in line with recent studies on other countries by Fowler (2015), Clark et al. (2015) and Jones et al. (2015)). The main added value of the scalable individualized neighbourhoods is their better ability to show differences across various spatial scales. By varying population counts, it becomes possible to compare segregation across much more spatial levels (and diverse size intervals) than is the case with administrative or statistical neighbourhoods which turns out to be useful to pinpoint segregation across different origin groups.

The factor analysis based on scalable individualized neighbourhoods showed that the relationship between segregation and spatial scale is much more specific and nuanced than can be shown by only using administrative units. The factor analysis on administrative units did not indicate large variations across the three included spatial scales. If at all, concentrations at the municipality level differed slightly from those at the neighbourhood and borough level. The scalable individualized neighbourhoods approach resulted in a typology of neighbourhoods where in one neighbourhood type high scores at smaller scales coincide with low scores at larger scales, while in other types concentrations are stable across different scales.

Both the factor analyses and the comparison of isolation index scores show that the segregation levels of the four non-western migrant origin groups in the Netherlands vary across the eleven individualized neighbourhoods. The spatial scale at which segregation is strongest or weakest is also strongly group-specific. Whereas the concentration of the Surinamese origin group is relatively stable across all spatial scales, people of Moroccan and Turkish origin are most strongly segregated at the smallest and medium-sized scales and much less at larger scales. The segregation of the Antillean origin group is a clear example of micro-level segregation.

These differences in segregation levels across scale can be explained by looking at their geographical patterns. The main Surinamese clusters are largely restricted to one large borough in Amsterdam and to a lesser degree in other large cities in the Randstad area. The Turkish, Moroccan and Antillean origin groups are found in more, albeit specific, larger and medium-sized municipalities, both within and outside the Randstad region. Within these municipalities, they are clustered in only a few administrative neighbourhoods and often even in specific sections of these districts.

The isolation index scores for all four origin groups confirm this picture: index scores based on all small and medium-sized individualized neighbourhoods are higher than those found for administrative neighbourhoods. These findings suggest that there is quite large heterogeneity within administrative districts. Even within moderately segregated administrative neighbourhoods, there may be small sections where the majority of the population belongs to one single group. This within-neighbourhood variation may be overlooked or underestimated when only studying segregation for administrative neighbourhoods.

Of course, using scalable individualized neighbourhoods also has limitations. Some administrative or statistical borders are drawn for a good reason, for example if they overlap with natural or physical barriers such as rivers, railroads or highways. EquiPop cannot control for such physical or natural barriers, which may therefore cut right through individualized neighbourhoods. Another potential disadvantage of using fixed population counts may be that different individualized neighbourhoods of the same population size have large differences in terms of surface, depending on population density. In sparsely populated rural areas, the distance covered for finding the same number of nearest neighbours is generally much larger than in densely populated urban neighbourhood. Also using a fixed distance radius rather than fixed population counts would not solve dependence on population density, since this would result in large differences in population count between districts with the same distance radius.

The overall conclusion of this study is that segregation is indeed manifested differently across spatial scales, and this relationship is complex and also group-specific. It is therefore problematic to use only one level of resolution in segregation research. Individualized neighbourhoods are a useful addition to current segregation research, since they seem better suited for studying a multiscalar phenomenon than static units, which are predefined on criteria not necessarily related to segregation.

As long as geocoded population register data are available, the method can be applied in the same way across different regional or national contexts. A next step would therefore be to directly compare segregation results between different countries. Although this study focused specifically on the Netherlands, ethnic segregation is certainly not a typical Dutch phenomenon. Although this may require a focus on broader migrant categories due to the specific migrant stocks across countries, it would be interesting to see whether similar outcomes also exist for other countries.

Another future step should be to study neighbourhood effects related to ethnic segregation for individualized neighbourhoods, both in the Dutch context, but also in an internationally comparative setting. Urban policies aimed at decreasing socio-spatial inequalities are generally targeting specific administrative neighbourhoods, but as we have seen the strongest levels of segregation occur also at spatial scales way smaller, or larger, than administrative neighbourhoods. Often such policies include urban regeneration programmes in which concentrations of social housing are replaced by a more diverse housing stock or reforms in allocation systems for social rented dwellings (Van Kempen and Bolt 2009; Andersson et al. 2010; Galster 2012). The main aim of such policies is to create socially mixed neighbourhoods, which are expected to provide better opportunities for integration. Still, empirical evidence on neighbourhood effects and the effectiveness of its countering policies is mixed, which according to Andersson and Malmberg (2015) may be due to the way neighbourhoods are defined. Given the relatively large size and ethnic heterogeneity of urban administrative neighbourhoods in the Netherlands, it is likely that focusing on neighbourhood effects on different spatial scales would result in different outcomes. Perhaps the context of an administrative neighbourhood does not influence one’s personal life chances, but living in highly segregated pockets *within* such neighbourhoods has a stronger effect. For other groups, however, focusing on areas larger than administrative neighbourhoods may be more relevant. An interesting avenue for future research in this respect is also to study which areas people *perceive* to be their neighbourhood. This is highly subjective and will require qualitative research. Although this has not yet been tested on Dutch data, the fact that migrant origin groups are concentrated at different spatial scales justifies studying neighbourhood effects for individualized neighbourhoods in future research.

## Appendix

**Table 2:** Factor loadings and explained variance of factor analysis based on administrative units

Factors based on administrative units						Explained variance (%)
*Factor 1: Surinamese and Antillean clusters*						35.4
	Native Dutch	Moroccan	Turkish	Surinamese	Antillean	
Neighbourhood	− 0.521	0.047	0.071	0.848	0.73	
Borough	− 0.563	0.135	0.154	0.864	0.803	
Municipality	− 0.626	0.31	0.304	0.87	0.861	
*Factor 2: Turkish clusters*						21.5
	Native Dutch	Moroccan	Turkish	Surinamese	Antillean	
Neighbourhood	− 0.561	0.362	0.907	0.126	0.197	
Borough	− 0.516	0.299	0.932	0.135	0.23	
Municipality	− 0.302	0.004	0.701	0.043	0.15	
*Factor 3: Moroccan clusters*						19.3
	Native Dutch	Moroccan	Turkish	Surinamese	Antillean	
Neighbourhood	− 0.426	0.778	0.17	0.076	0.104	
Borough	− 0.427	0.862	0.161	0.089	0.149	
Municipality	− 0.49	0.834	0.225	0.217	0.213	

**Table 3:** Factor loadings and explained variance of factor analysis based on individualized units

Factors based on individualized neighbourhoods						Explained variance (%)
*Factor 1: Surinamese clusters*						21.8
*k*-levels	Native Dutch	Moroccan	Turkish	Surinamese	Antillean	
50	− 0.338	0.113	0.106	0.827	0.197	
100	− 0.35	0.121	0.111	0.868	0.224	
200	− 0.359	0.133	0.118	0.89	0.264	
400	− 0.365	0.143	0.12	0.897	0.304	
800	− 0.377	0.154	0.12	0.896	0.343	
1600	− 0.39	0.164	0.121	0.89	0.382	
3200	− 0.401	0.173	0.121	0.882	0.42	
6400	− 0.413	0.181	0.122	0.871	0.449	
12,800	− 0.428	0.194	0.128	0.858	0.471	
25,600	− 0.439	0.209	0.136	0.834	0.484	
51,200	− 0.451	0.235	0.151	0.795	0.494	
*Factor 2: Turkish clusters*						16.5
*k*-levels	Native Dutch	Moroccan	Turkish	Surinamese	Antillean	
50	− 0.35	0.222	0.804	0.135	0.072	
100	− 0.367	0.235	0.848	0.142	0.092	
200	− 0.375	0.251	0.876	0.143	0.118	
400	− 0.381	0.263	0.887	0.143	0.145	
800	− 0.384	0.268	0.883	0.139	0.167	
1600	− 0.377	0.262	0.863	0.132	0.178	
3200	− 0.359	0.246	0.828	0.121	0.18	
6400	− 0.33	0.221	0.777	0.108	0.172	
12,800	− 0.288	0.189	0.707	0.089	0.153	
25,600	− 0.237	0.147	0.626	0.064	0.126	
51,200	− 0.178	0.098	0.534	0.038	0.097	
*Factor 3: Moroccan clusters*						16
*k*-levels	Native Dutch	Moroccan	Turkish	Surinamese	Antillean	
50	− 0.346	0.813	0.225	0.14	0.071	
100	− 0.364	0.856	0.24	0.149	0.088	
200	− 0.372	0.88	0.254	0.161	0.112	
400	− 0.374	0.885	0.261	0.168	0.132	
800	− 0.371	0.875	0.26	0.17	0.144	
1600	− 0.361	0.845	0.25	0.166	0.148	
3200	− 0.34	0.8	0.231	0.155	0.141	
6400	− 0.311	0.744	0.204	0.142	0.125	
12,800	− 0.273	0.677	0.171	0.124	0.103	
25,600	− 0.231	0.6	0.133	0.105	0.074	
51,200	− 0.193	0.517	0.092	0.098	0.051	
*Factor 4: Antillean clusters at larger scales*						9.2
*k*-levels	Native Dutch	Moroccan	Turkish	Surinamese	Antillean	
50	− 0.098	0.055	0.054	− 0.011	0.066	
100	− 0.119	0.057	0.059	0.015	0.136	
200	− 0.154	0.059	0.066	0.068	0.263	
400	− 0.193	0.063	0.076	0.125	0.396	
800	− 0.236	0.073	0.088	0.181	0.52	
1600	− 0.275	0.085	0.102	0.23	0.621	
3200	− 0.303	0.091	0.112	0.27	0.683	
6400	− 0.321	0.092	0.118	0.3	0.719	
12,800	− 0.339	0.092	0.124	0.33	0.74	
25,600	− 0.348	0.084	0.128	0.349	0.74	
51,200	− 0.337	0.074	0.129	0.35	0.701	
*Factor 5: Low diversity at small scales, clusters non*-*western at larger scales*						9.1
*k*-levels	Native Dutch	Moroccan	Turkish	Surinamese	Antillean	
50	− 0.059	− 0.051	− 0.062	0.025	0.077	
100	− 0.074	− 0.031	− 0.044	0.032	0.067	
200	− 0.107	0.02	0.004	0.044	0.049	
400	− 0.151	0.084	0.066	0.062	0.034	
800	− 0.211	0.167	0.144	0.086	0.035	
1600	− 0.283	0.268	0.235	0.114	0.054	
3200	− 0.361	0.377	0.341	0.144	0.085	
6400	− 0.441	0.485	0.456	0.173	0.126	
12,800	− 0.515	0.585	0.564	0.202	0.164	
25,600	− 0.563	0.654	0.64	0.221	0.19	
51,200	− 0.557	0.66	0.646	0.229	0.195	
*Factor 6: Antillean clusters at small scales*						8.8
*k*-levels	Native Dutch	Moroccan	Turkish	Surinamese	Antillean	
50	− 0.26	0.079	0.057	0.232	0.843	
100	− 0.263	0.086	0.066	0.235	0.879	
200	− 0.25	0.09	0.077	0.227	0.837	
400	− 0.232	0.093	0.087	0.212	0.752	
800	− 0.211	0.092	0.093	0.192	0.647	
1600	− 0.186	0.083	0.096	0.17	0.536	
3200	− 0.161	0.073	0.096	0.144	0.44	
6400	− 0.137	0.063	0.095	0.119	0.364	
12,800	− 0.115	0.054	0.097	0.09	0.297	
25,600	− 0.092	0.042	0.096	0.061	0.239	
51,200	− 0.071	0.033	0.09	0.041	0.188	
*Factor 7: Low shares non*-*western migrants*						8
*k*-levels	Native Dutch	Moroccan	Turkish	Surinamese	Antillean	
50	0.654	− 0.151	− 0.181	− 0.134	− 0.108	
100	0.669	− 0.157	− 0.185	− 0.141	− 0.121	
200	0.663	− 0.155	− 0.18	− 0.145	− 0.13	
400	0.642	− 0.151	− 0.168	− 0.146	− 0.139	
800	0.607	− 0.141	− 0.15	− 0.144	− 0.143	
1600	0.566	− 0.128	− 0.127	− 0.141	− 0.145	
3200	0.529	− 0.116	− 0.1	− 0.136	− 0.146	
6400	0.497	− 0.106	− 0.073	− 0.132	− 0.147	
12,800	0.467	− 0.105	− 0.05	− 0.132	− 0.145	
25,600	0.449	− 0.116	− 0.043	− 0.137	− 0.15	
51,200	0.448	− 0.154	− 0.064	− 0.149	− 0.169	

## Footnotes

1 The country of birth is known for all persons registered in SSD, and the country of birth of the parents is known for all but 2386 persons. In line with definitions used by Statistics Netherlands, those with two parents born in the Netherlands are categorized as native Dutch while those who are born abroad are labelled as first-generation migrants. ‘Second-generation descendants of migrants’ are born in the Netherlands themselves, but have at least one parent who was born abroad. The mother’s country of birth determines origin. In case the mother was born in the Netherlands, the father’s country of birth determines their origin.

2 The central grid with five inhabitants is taken as the starting point (a). EquiPop then starts looking for nearest neighbours in the adjacent grid cells (b) and does this exactly the same way from each grid cell. It stops looking for nearest neighbours after the grid cell in which the 50th person was found (c), but includes all people living in that final grid, in order to avoid omitting persons randomly. The number of grids needed in order to reach the k-number of nearest neighbours of course depends on the population density of the region under study: in densely populated areas such as large cities, generally fewer grids will be needed to reach the same number of neighbours than in rural areas with lower population density.
